# Altered Brain Network in Amyotrophic Lateral Sclerosis: A Resting Graph Theory-Based Network Study at Voxel-Wise Level

**DOI:** 10.3389/fnins.2016.00204

**Published:** 2016-05-10

**Authors:** Chaoyang Zhou, Xiaofei Hu, Jun Hu, Minglong Liang, Xuntao Yin, Lin Chen, Jiuquan Zhang, Jian Wang

**Affiliations:** ^1^Department of Radiology, Southwest Hospital, Third Military Medical UniversityChongqing, China; ^2^Department of Neurology, Southwest Hospital, Third Military Medical UniversityChongqing, China

**Keywords:** amyotrophic lateral sclerosis, degree centrality, graph theory-based network, resting state, magnetic resonance imaging

## Abstract

Amyotrophic lateral sclerosis (ALS) is a rare degenerative disorder characterized by loss of upper and lower motor neurons. Neuroimaging has provided noticeable evidence that ALS is a complex disease, and shown that anatomical and functional lesions extend beyond precentral cortices and corticospinal tracts, to include the corpus callosum; frontal, sensory, and premotor cortices; thalamus; and midbrain. The aim of this study is to investigate graph theory-based functional network abnormalities at voxel-wise level in ALS patients on a whole brain scale. Forty-three ALS patients and 44 age- and sex-matched healthy volunteers were enrolled. The voxel-wise network degree centrality (DC), a commonly employed graph-based measure of network organization, was used to characterize the alteration of whole brain functional network. Compared with the controls, the ALS patients showed significant increase of DC in the left cerebellum posterior lobes, bilateral cerebellum crus, bilateral occipital poles, right orbital frontal lobe, and bilateral prefrontal lobes; significant decrease of DC in the bilateral primary motor cortex, bilateral sensory motor region, right prefrontal lobe, left bilateral precuneus, bilateral lateral temporal lobes, left cingulate cortex, and bilateral visual processing cortex. The DC's z-scores of right inferior occipital gyrus were significant negative correlated with the ALSFRS-r scores. Our findings confirm that the regions with abnormal network DC in ALS patients were located in multiple brain regions including primary motor, somatosensory and extra-motor areas, supporting the concept that ALS is a multisystem disorder. Specifically, our study found that DC in the visual areas was altered and ALS patients with higher DC in right inferior occipital gyrus have more severity of disease. The result demonstrated that the altered DC value in this region can probably be used to assess severity of ALS.

## Introduction

Amyotrophic lateral sclerosis (ALS) is a rare degenerative disorder selectively affecting upper and lower motor neurons, culminating in respiratory insufficiency and death after 3–5 years (Beghi et al., 2006). The cause of ALS remains unknown (Orrell, 2007). Functional MRI (fMRI) has provided an important tool to study ALS–related cortical function and reorganizations. In small samples of patients with ALS, the fMRI tasks of limb movements have demonstrated that regional modifications of cerebral activation involved extensive cortical regions including the primary motor cortex, supplementary motor areas, the premotor cortex, the motor learning areas (basal ganglia and cerebellum), and extra-motor areas (e.g., the temporal and inferior parietal cortices; Chiò et al., 2014). However, the task fMRI in ALS may have a large variability in task performance because of the disease induced peripheral weakness (Pradat and El Mendili, 2014). The resting-state functional MRI (rsfMRI) techniques avoid potential performance because of being independent of task demand. Several rsfMRI studies of ALS reported significantly decreased functional connectivity within the sensorimotor network (Mohammadi et al., 2009; Jelsone-Swain et al., 2010; Tedeschi et al., 2012; Fekete et al., 2013; Zhou et al., 2013; Chiò et al., 2014) and in brain networks related to cognition and behavior, in keeping with the altered motor and extramotor structural connectivity. Other studies have identified regions of increased functional connectivity, including somatosensory and extra-motor areas (Verstraete et al., 2010; Agosta et al., 2011, 2013; Douaud et al., 2011; Luo et al., 2012; Fekete et al., 2013; Zhou et al., 2013; Chiò et al., 2014; Zhou F. et al., 2014). As these studies are based on networkwise functional connectivity analysis using seed-based correlation analysis or independent component analysis, none of these studies fully characterize the brain's functional connectome of ALS.

Recently, the graph theory-based network analysis was applied to characterize functional connectivity within the whole-brain functional connectome (Rubinov and Sporns, 2010; Telesford et al., 2011). The voxel-wise degree centrality (DC), a class of graph theory-based network measures assessing functional importance degree, has been widely used to detect changes in resting-state functional networks (Cole et al., 2010; Wang et al., 2010; Fransson et al., 2011; Lord et al., 2011; Di Martino et al., 2013; Kullmann et al., 2014; Zhou Y. et al., 2014). When a node has numerous direct connections to other nodes, it will have a high DC. It represents the most local and directly quantifiable centrality measure. Different from seed-based approaches or independent component analysis, this graph-based measure of network organization can characterize the functional relationships of a given node(voxel) within the entire connectivity matrix of full-brain functional connectome rather than represent the relationships with specific nodes or networks (Tomasi and Volkow, 2011; Zuo et al., 2012). As such, DC analysis allow us to capture the complexity of the functional connectome (Zuo et al., 2012). Furthermore, the exploration of voxel-wise DC allowed us to identify the brain regions that may be involved by ALS without requiring a priori selection of nodes or networks of interest (Zhou Y. et al., 2014).

In this study, we explored the full-brain functional networks using rsfMRI data obtained in 43 sporadic ALS patients and 44 health control (HC). The voxel-wise network DC was analyzed. We hypothesize that ALS would alter the DC of multiple brain regions within the entire connectome compared with HC.

## Materials and methods

### Participants

Forty-three sporadic ALS patients (30 definite, 13 probable) were recruited consecutively from the Department of Neurology at Southwest Hospital in Chongqing and were diagnosed according to the El Escorial criteria (Brooks et al., 2000). Inclusion criteria: a diagnosis of definite or probable ALS and right-handedness. Exclusion criteria were as follows: (1) family history of motor neuron diseases; (2) clinical diagnosis of frontotemporal dementia (Neary et al., 1998); (3) history of brain injury, epilepsy, or a neurologic disease; (4) significant respiratory failure (forced vital capacity below 70); and (5) cognitive impairment as determined by Montreal Cognitive Assessment (MoCA) score < 26 (Nasreddine et al., 2005). Disease severity was assessed using the ALS Functional Rating Scale-revised (ALSFRS-r; Cedarbaum et al., 1999). Forty-four age- and sex-matched healthy volunteers, with no history of neurological or psychiatric disorders and a normal neurological examination, were recruited from the local community served as HC. All the participants were right-handed investigated by Edinburgh inventory (Oldfield, 1971) and all MRI scans were visually inspected by a radiologist to rule out major neuropathology such as tumor, stroke, or advanced white matter disease.

The study was approved by the local ethical committee. All subjects provided written informed consent before enrollment.

### MRI acquisition

The MRI data were acquired with a 3.0T Siemens Tim Trio whole-body MRI system (Siemens Medical Solutions, Erlangen, Germany) in the Southwest Hospital, Chongqing, China. Whole-brain functional scans were collected in 36 axial slices by using an echo-planar imaging (EPI) sequence with the following settings: TR = 2000 ms, TE = 30 ms, flip angle = 90°, FOV = 192 × 192 mm, slices = 36, in-plane matrix = 64 × 64, thickness = 3 mm, voxel size = 3.0 × 3.0 × 3.0 mm. For each subject, a total of 240 volumes were acquired, resulting in a total scan time of 480s. Three dimensional T1-weighted anatomical images were acquired in a sagittal orientation using the following volumetric 3D magnetization-prepared rapid gradient–echo (MP-RAGE) sequence (TR = 1900 ms, TE = 2.52 ms, flip angle = 9°, slice thickness = 1 mm, slices, 176, FOV = 256 × 256 mm, matrix size = 256 × 256 and voxel size = 1 × 1 × 1 mm) on each subject. All subjects were instructed simply to rest with their eyes closed, not to think of anything in particular, and not to fall asleep.

### Data preprocessing

All preprocessing steps were performed using the Data Processing Assistant for Resting-State fMRI (DPARSF2.3, http://www.restfmri.net), which is based on the Statistical Parametric Mapping (SPM8) program (http://www.fil.ion.ucl.ac.uk/spm) and the Resting-State fMRI Data Analysis Toolkit (REST1.8, http://www.restfmri.net). Prior to preprocessing, the first 10 volumes were discarded to allow for signal stabilization due to instability of the initial MRI signal and adaptation of participants to the circumstance. The remaining volumes of each subject were corrected for the acquisition differences between slices. The resulting images were then realigned to correct for small movements that occurred between scans. Based on the recorded motion correction estimates, the subjects with more than 1.5 mm maximum displacement in any of the x, y, or z directions or more than 1.5° of angular rotation about any axis for any of the 230 volumes were excluded from the study. Based on these criteria, four ALS and five HCs were excluded from the analyses. Individual 3D T1-weighted structural images were co-registered to the mean of the realigned EPI images. The transformed structural images were then separated into white matter, gray matter, and cerebrospinal fluid. The Diffeomorphic Anatomical Registration Through Exponentiated Lie (DARTEL) algebra tool (Ashburner, 2007) was applied to compute the transformations from individual native space to MNI space.

As rsfMRI measures have been shown to be sensitive to micro-head motions (Yan et al., 2013a), the Friston 24-parameter model was applied to regress head motion effects out of the realigned data (Satterthwaite et al., 2013; Yan et al., 2013a). Considering measures of voxel-wise differences in motion in its derivation, we further regressed the Jenkinson's mean frame-wise displacement (FD) (a measure of the micro-head motion; Yan et al., 2013b) of each subject, as recommended in a previous study (Yan et al., 2013a).

To further reduce the effects of confounding factors, the signals from the white matter and cerebrospinal fluid, the mean time series of all voxels across the whole brain and linear and quadratic trends were removed from the data with linear regression (Yan et al., 2013b). The resulting maps were then registered into MNI space with 3 mm^3^ cubic voxels using the transformation information acquired from DARTEL. A temporal filter (0.01–0.08 Hz) was applied to reduce low frequency drifts and high frequency physiological noise.

### Degree centrality calculation

To exclude artifactual correlations from non-gray matter voxels, the voxel-wise centrality analyses were restricted to a predefined gray matter mask that included tissue with gray matter probabilities more than 20% as previously described (Zuo et al., 2012; Zhou Y. et al., 2014). The gray matter tissue probability template was published as a part of tissue priors in SPM8. Within the study mask, individual network centrality maps were created in a voxel-wise style. First, the preprocessed functional data sets were subjected to voxel-based whole-brain correlation analysis. The time course of each voxel within the gray matter mask from each participant was correlated with the time course of every other voxel, which generated a correlation matrix. An undirected adjacency matrix was then obtained by thresholding each correlation at *r* > 0.25 (Zuo et al., 2012; Yan et al., 2013a,b; Zhou Y. et al., 2014). Then, the DC was computed as the sum of the weights of the significant correlations (weighted) or the number of significant connections (binarized) for each voxel (Zuo et al., 2012; Zhou Y. et al., 2014). Finally, by subtracting the mean DC across the entire brain and then dividing by the standard deviation of the whole-brain DC, these individual-level voxel-wise DC maps were standardized into a z-score (Zuo et al., 2012; Yan et al., 2013b). A smoothing kernel of 4 mm was applied.

### Statistical analysis

Two samples *t*-tests were performed to examine the differences between the DC measures of ALS group and HC group by REST1.8 (Song et al., 2011). Statistical significance was set at a voxel-wise *p* < 0.01 in conjunction with cluster wise AlphaSim (rmm = 5, clusters = 18) to correct for multiple comparisons. In AlphaSim, Monte Carlo permutation simulations are used to estimate the null distribution (Cox and Hyde, 1997; Jadach et al., 2001). Generally, the voxel-wise intensity threshold was set at *p* < 0.01 and a cluster-level threshold of 405 mm^3^ (which was 18 voxels) was calculated using the Monte Carlo simulation in AFNI (10,000 iterations, 61 × 73 × 61 dimensions, 3 × 3 × 3 m^3^, 4-mm smoothness), in which case the probability of a type I error was less than 0.01.

At last, pearson correlations (two-sided) were calculated between ALSFRS-r score and z-score of altered DC's areas in ALS group with each patient's age and gender as covariates.

## Results

The demographic and clinical characteristics of participants analyses are summarized in Table 1. There was no significant difference in age and gender between the ALS group and HC group.

**Table 1:** **Demographic Data of the participants**.

**Group**	**ALS^a^**	**Controls**	***p* value^c^**
Age (years)	49.05 ± 8.07529–71)	51.00 ± 10.576 (24–65)	0.386
Male: female	26:13	21:18	0.247
El Escorial criteria (probable/definite)	12/27	–	–
Disease duration (months)	17.77 ± 13.0012–71)	–	–
ALSFRS-R^a^	36.18 ± 6.472 (16–44)	–	–
Disease progression rate^b^	0.9210 ± 0.70944 (0.17–3.14)	–	–

a ALS, amyotrophic lateral sclerosis; ALSFRS_R, ALS Functional Rating Scale_Revised.

b Disease progression rate = (48–ALSFRS_R)/Disease duration.

c Statistical significance was set at p < 0.05.

**Figure 1:**
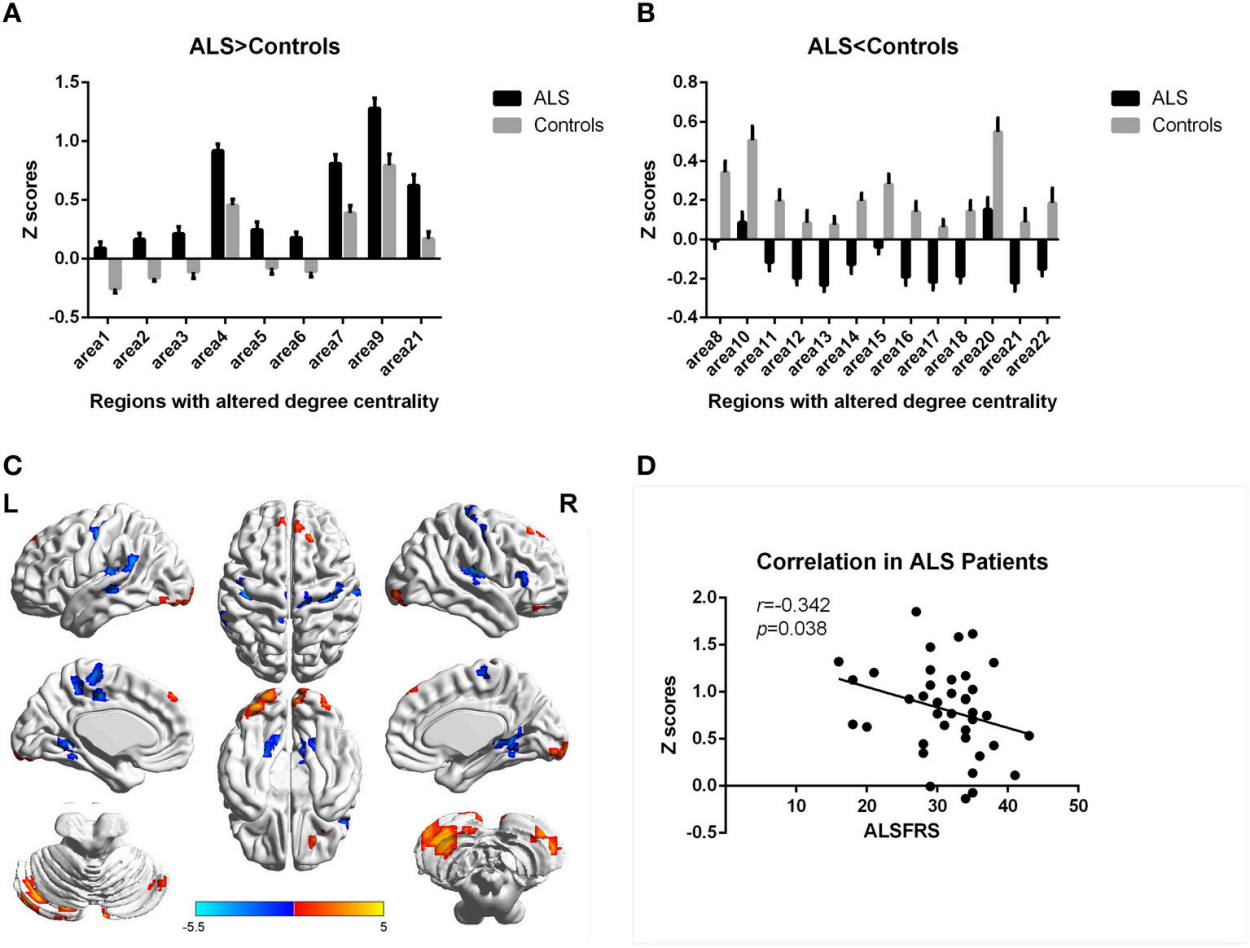
**Regions with altered degree centrality in ALS patients compared with healthy controls in weighted networks**. **(A,B)** represents the regions with increase/decrease degree centrality in ALS patients in bar graphs (group mean Z scores and standard errors of the mean). The red areas in **(C)** show the regions with increased degree centrality, and the blue areas show the regions with decreased degree centrality. The color bar shows the T values. **(D)** shows the negative correlation between DC's z-scores in weighted networks of Right inferior Occipital gyrus (area7, BA 18) and the ALSFRS-r scores in ALS group with the age and gender as covariates.

**Figure 2:**
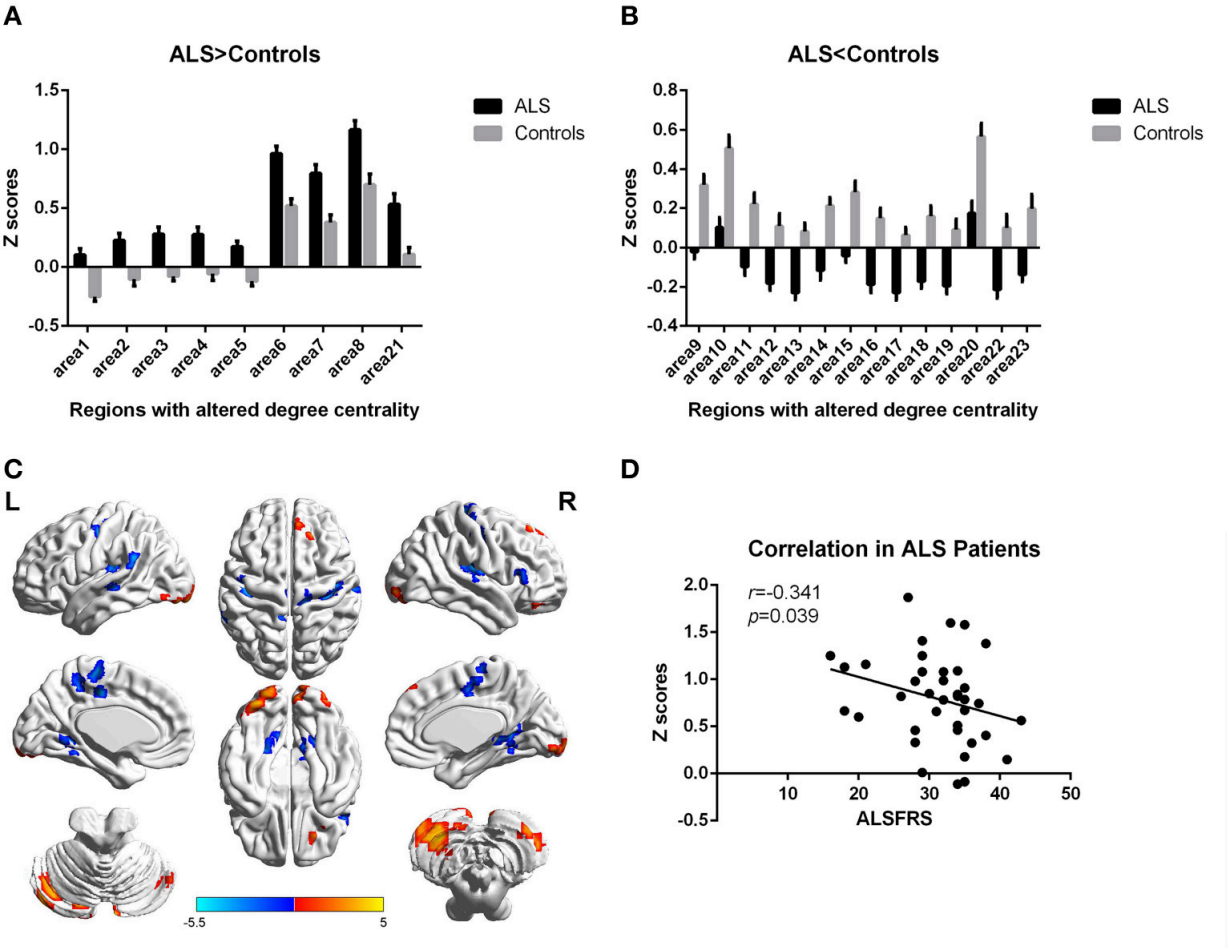
**Regions with altered degree centrality in ALS patients compared with healthy controls in binarized networks**. **(A,B)** represents the regions with increase/decrease degree centrality in ALS patients in bar graphs (group mean Z scores and standard errors of the mean). The red areas in **(C)** show the regions with increased degree centrality, and the blue areas show the regions with decreased degree centrality. The color bar shows the T values. **(D)** shows the negative correlation between DC's z-scores in binarized networks of Right inferior Occipital gyrus (area7, BA 18) and the ALSFRS-r scores in ALS group with the age and gender as covariates.

In the present work, centrality analyses were performed for both binary and weighted graphs representing the functional connectome. Compared to the HC group in the weighted DC, significant increases of DC in ALS group were found in the left cerebellum posterior lobes, bilateral cerebellum crus, bilateral occipital poles (Brodmann area(BA) 18/19), right orbital frontal lobe(BA11) and bilateral prefrontal lobes (BA8/9; Figures 1A,C; Table 2); significant decreases of DC in ALS group were found in the bilateral primary motor cortex(BA4/6), bilateral sensory motor region(BA3/5), right prefrontal lobe(BA45), left precuneus(BA5), bilateral lateral temporal lobes(BA48), left middle cingulate cortex(BA23), and bilateral visual processing cortex(BA17/18/37; Figures 1B,C; Table 3). The findings obtained from the binarized networks were similar to those obtained from the weighted networks (Figures 2A–C; Tables 4, 5). Correlation analysis shows that DC's z-scores of Right inferior Occipital gyrus (area7, BA 18) is negative correlated with the ALSFRS-r scores in both the binarized and weighted networks of ALS group (Figures 1D, 2D).

**Table 2:** **Regions with increase degree centrality in patients with ALS compared with healthy controls in weighted networks**.

**Areas**	**Voxel size^a^**	**Voxels structure (BA)^b^**	**Peak MNI coordinate**^**b**^			**Peak intensity (T value)**	**Peak MNI coordinate region**
			**X**	**Y**	**Z**		
Area 1	190		–27	–69	–48	4.3181	Left Cerebellum Posterior Lobe7
Area 2	97		–3	–87	–36	4.6095	Left Cerebellum Crus 2
Area 3	38		33	–69	–42	3.4569	Right Cerebellum Crus 2
Area 4	19		–15	–75	–27	3.6728	Left CerebellumCrus 1
Area 5	107	BA18/19	–33	–87	–18	4.044	Left Lingual gyrus
Area 6	35	BA11	24	42	–21	3.4221	Right Middle Orbital Frontal gyrus
Area 7	22	BA18/19	36	–96	–9	3.6865	Right inferior Occipital gyrus
Area 9	19	BA18	9	–94	–12	3.087	Right Lingual gyrus
Area 19	49	BA8/9	12	45	54	3.8211	Superior Frontal gyrus

a Statistical significance was set at a voxel-wise p < 0.01, in conjunction with cluster wise AlphaSim (rmm = 5, clusters = 18) to correct for multiple comparisons.

b BA, Brodmann area; MNI, Montreal Neurological Institute.

**Table 3:** **Regions with decrease degree centrality in patients with ALS compared with healthy controls in weighted networks**.

**Areas**	**Voxel size^a^**	**Voxels structure (BA)^b^**	**Peak MNI coordinate**^**b**^			**Peak intensity (T value)**	**Peak MNI coordinate region**
			**X**	**Y**	**Z**		
Area 8	49	BA18/37	–21	–51	–3	–4.3451	Left Lingual gyrus, extent to fusiform gyrus
Area 10	89	BA17/18/37	18	–48	3	–4.4131	Right Calcarine gyrus, extent to Lingual gyrus, and fusiform gyrus
Area 11	21	BA21	–54	–27	–3	–4.3048	Left Middle Temporal
Area 12	18	BA45	60	27	6	–3.5382	Right Triangular Part of inferior Frontal gyrus
Area 13	119	BA48	33	–18	21	–4.7377	Right Insula gyrus
Area 14	47	BA48	–36	–27	12	–4.4054	Left Heschl gyrus
Area 15	25	BA48	–66	–48	24	–4.2531	Left SupraMarginal gyrus
Area 16	175	BA3/4	42	–12	27	–4.7412	Right Precentral gyrus, extent to Postcentral gyrus
Area 17	32	BA23	–9	–21	45	–3.9378	Left Middle Cingulum gyrus
Area 18	69	BA3/6	–45	–15	51	–5.1545	Left Postcentral gyrus
Area 20	36	BA5	–9	–39	60	–4.3082	Left Precuneus gyrus
Area 21	24	BA4	–6	–27	60	–3.8403	Left Paracentral gyrus
Area 22	37	BA4	15	–21	72	–3.7799	Right Precentral gyrus

a Statistical significance was set at a voxel-wise p < 0.01, in conjunction with cluster wise AlphaSim (rmm = 5, clusters = 18) to correct for multiple comparisons.

b BA, Brodmann area; MNI, Montreal Neurological Institute.

**Table 4:** **Regions with increase degree centrality in patients with ALS compared with healthy controls in the binarizednetworks**.

**Areas**	**Voxel size^a^**	**Voxels structure (BA)^b^**	**Peak MNI coordinate**^**b**^			**Peak intensity (T value)**	**Peak MNI coordinate region**
			**X**	**Y**	**Z**		
Area 1	194		–27	–69	–48	4.4222	Left Cerebellum Posterior Lobe7
Area 2	41		33	–69	–42	3.5008	RightCerebellumCrus
Area 3	108		–3	–87	–36	4.6621	Left CerebellumCrus 2
Area 4	18		–15	–75	–27	3.679	Left CerebellumCrus 1
Area 5	37	BA11	24	42	–21	3.8082	Right Middle Orbital Frontal gyrus
Area 6	85	BA18/19	–24	–96	–18	3.997	Left Lingual gyrus
Area 7	25	BA18	36	–96	–9	3.7465	Right inferior Occipital gyrus
Area 8	21	BA18	12	–102	–6	3.0777	Right Lingual gyrus
Area 21	38	BA8/9	12	45	54	3.7879	Superior Frontal gyrus

a Statistical significance was set at a voxel-wise p < 0.01, in conjunction with cluster wise AlphaSim (rmm = 5, clusters = 18) to correct for multiple comparisons.

b BA, Brodmann area; MNI, Montreal Neurological Institute.

**Table 5:** **Regions with decrease degree centrality in patients with ALS compared with healthy controls in the binarized networks**.

**Areas**	**Voxel size^a^**	**Voxels structure (BA)^b^**	**Peak MNI coordinate**^**b**^			**Peak intensity (T value)**	**Peak MNI coordinate region**
			**X**	**Y**	**Z**		
Area 9	42	BA18/37	–21	–51	–3	–4.3062	Left Lingual gyrus, extent to fusiform gyrus
Area 10	81	BA17/18/37	18	–48	3	–4.3412	Right Calcarine gyrus, extent to Lingual gyrus, and fusiform gyrus
Area 11	19	BA22	–54	–27	–3	–4.2576	Left Middle Temporal
Area 12	20	BA45	60	27	6	–3.6089	Right Triangular Part of inferior Frontal gyrus
Area 13	115	BA48	33	–18	21	–4.757	Right Insula gyrus
Area 14	45	BA48	–36	–27	12	–4.3269	Left Heschl gyrus
Area 15	22	BA48	–66	–48	24	–4.2694	Left SupraMarginal gyrus
Area 16	166	BA3/4/6	42	–12	27	–4.5985	Right Precentral gyrus, extent to Postcentral gyrus
Area 17	42	BA23	–9	–21	45	–4.1167	Left Middle Cingulum gyrus
Area 18	67	BA3/4/6	–45	–15	51	–5.2347	Left Postcentral gyrus extent to Precentral gyrus
Area 19	19	BA4/23	6	–18	57	–3.7851	Right Supplement Motor Area, extent to Precentral gyrus
Area 20	39	BA5	–9	–39	60	–4.3001	Left Precuneus gyrus
Area 22	24	BA4	–6	–27	60	–3.8749	Left Paracentral gyrus
Area 23	37	BA4	15	–21	72	–3.8176	Right Precentral gyrus

a Statistical significance was set at a voxel-wise p < 0.01, in conjunction with cluster wise AlphaSim (rmm = 5, clusters = 18) to correct for multiple comparisons.

b BA, Brodmann area; MNI, Montreal Neurological Institute.

## Discussion

Intrinsic functional connectivity provides a powerful and unique tool for the examination of the organization of the human brain (Buckner et al., 2013). To our knowledge, this is the first study to conduct a voxel-based analysis of brain functional network during the resting state on a whole brain scale in ALS patients with DC measurement, and our results revealed that ALS patients showed abnormal network DC in multiple brain regions. Our results suggest that the DC measurement of full-brain functional networks could be useful to characterize the pathophysiology of ALS. Voxel-wise whole-brain analysis of functional connectivity, as is conducted in our study, allows for unbiased mapping of ALS-associated functional abnormalities which offers advantages over the region-based analysis of functional connectivity requiring a priori spatial demarcations (Zhou Y. et al., 2014). Furthermore, compared with previous functional connectivity analysis using seed-based correlation analysis or independent component analysis, the voxel-based network DC measurement directly and quantifiably reflects the change between multiple brain regions system and the whole brain functional network connectome.

Specifically, our study found that DC in the visual areas was altered in ALS patients: significant decreases DC in the bilateral anterior visual processing regions, significant increases DC in the bilateral caudate occipital poles. Previous study by detecting spontaneous low-frequency fluctuations (LFF) of blood oxygen level–dependent signals of rfMRI showed decreased ALFF in the visual processing areas: the inferior occipital lobe and fusiform gyri (Luo et al., 2012). This result and our findings suggest a disorder of visual process, which is in accordance with previous task-related fMRI studies (Lulé et al., 2007; Wang et al., 2013) and electrophysiological studies(Münte et al., 1998, 1999). A delayed and decreased response to visual stimuli was observed in sensory processing cortical areas in ALS patients by electrophysiological studies (Münte et al., 1998, 1999), and a previous longitudinal study identified progressive reduced activation of extrastriate regions over the duration of ALS (Lulé et al., 2007). Combining fMRI with structural MRI, investigators demonstrated the existence of a decreased response in secondary visual areas in ALS during visual stimulation, and significantly less white matter fiber tracts projecting to visual areas (Lulé et al., 2010). The decreases DC in visual processing areas strongly suggest that abnormalities in visual processing areas may be aroused by the demyelination of nerve fibers in the optic tract (Lulé et al., 2010). The increased DC in caudate visual areas (occipital pole) probably reflects a compensatory response due to disorder of visual process. Moreover, Correlation analysis shows that the DC's z-scores of right inferior occipital gyrus (BA18) was negative correlated with the ALSFRS-r scores in both the binarized and weighted networks of ALS group after eliminating the influence of age and gender, showing that ALS patient with higher DC in the area have more severity of disease. It demonstrated that increasing DC in right inferior occipital gyrus can probably be used to assess severity of ALS.

Our finding showed the decreased DC in the primary motor cortex and the sensory motor cortex, which represents that the sum of this region's functional connectivity with the entire connectome is reduced. The primary motor cortex is the hallmark area of impairment in ALS. Surface-based morphometry has showed cortical thinning of the motor cortex (Verstraete et al., 2010; Agosta et al., 2012; Mezzapesa et al., 2013). Previous rsfMRI studies of ALS reported significantly decreased functional connectivity within the sensorimotor network (Mohammadi et al., 2009; Jelsone-Swain et al., 2010; Tedeschi et al., 2012; Fekete et al., 2013; Zhou et al., 2013), which are in keeping with the altered motor and extra-motor functional connectivity (Luo et al., 2012; Agosta et al., 2013). In addition, neuronal loss in the motor cortex has also been demonstrated in previous PET studies and 1H-magnetic resonance spectroscopy study (Mitsumoto et al., 2007; Lombardo et al., 2009; Han and Ma, 2010; Pyra et al., 2010; Sudharshan et al., 2011; Chiò et al., 2014). Therefore, our findings are consistent with these neuroimaging studies and the ALS pathological feature.

In addition, our study also shows significant increases DC in the left cerebellum posterior lobes, bilateral cerebellum crus in ALS. Several neuropathological studies (Wang et al., 2012; Yue et al., 2015) and neuroimaging studies using voxel-based morphometry, diffusion-tensor imaging and functional MRI (Prell and Grosskreutz, 2013; Meoded et al., 2015) have shown evidence for involvement of the cerebellum in ALS. Our finding support the perspectives that recruitment of the cerebellum probably represents a compensatory response due to declining motor function and a loss of cortical inhibitory neuronal influence. However, this study didn't reveal significant correlation between ALSFR-s and altered DC of areas in the primary motor area, the sensory motor area and cerebellum, which may be owing to inclusion of patients with clinically homogeneous.

Moreover, our findings in the limbic system of the ALS patients, including the increased DC in right orbital frontal lobe and decreased DC in the temporal lobe probably reflect the dysfunctions of the emotional regulation. The depression, anxiety, apathy, personality disorders, and scarce impulse control have been described in up to 60% of ALS patients (An et al., 2013; Yuan et al., 2013; Xu et al., 2014). The previous fMRI studies about ALS patients in a series of brain regions broadly related to emotional functions have reported both reduced and enhanced responses (Lulé et al., 2007; Agosta et al., 2013; Passamonti et al., 2013; Wang et al., 2013; Chiò et al., 2014). In the other study, ALS patients displayed the dysfunctions of the limbic system in ALS patients and the interplay between emotional and motor control brain areas (Passamonti et al., 2013). These findings in the limbic system demonstrate that emotional dysregulation may underlie the inappropriate reactions to emotional stimuli and the abnormalities in social and behavior functioning in ALS patients (Sui et al., 2013; Vargas et al., 2013).

In the present study, we found increased DC in the bilateral superior frontal gyrus and right orbital frontal lobe, and decreased DC in left middle cingulate cortex and the left precuneus, which are crucial nodes of the default mode network (DMN; Buckner et al., 2008). Some researchers detecting an impaired DMN in both the posterior cingulate and inferior parietal cortices and the anterior cingulate and medial prefrontal cortices in ALS patients (Mohammadi et al., 2009), while others detected no such observations (Tedeschi et al., 2012). All these discordance across studies may be owing to methodological differences in the fMRI analysis or inclusion of patients with different clinical and neuropsychological features (Agosta et al., 2013). Future studies with neuropsychological assessments are needed to evaluate whether an association exists between abnormal network DC and behavioral or neuropsychology disturbances in ALS.

There are several limitations in this study. First, this study has a major limitation related to the small sample size and unbalanced characteristics of patients. Different onset and involvement of upper motor neuron, bulbar motor neurons and spinal motor neurons at an early stage in ALS represents different endophenotypes of the disorder, and there is a need to recruit a larger group of ALS patients and clinically homogeneous group for long-term longitudinal observation in future studies. Identification of subgroups depending on the onset clinical symptoms would be useful to discriminate putative central differentiation patterns of altered networks. Second, lack of objective assessment of behavior or neuropsychology in ALS patients hampers our interpretations of the results. Further investigations should be focused on the relationship between neuropsychological deficits and abnormal network DC in the extra-motor areas.

In conclusion, the present work shows the importance of DC in exploring the whole-brain functional network reorganization in ALS, whose neurobiological mechanisms are only partially known. Overall, our findings confirm that the abnormal network DC in ALS patients was located in multiple brain regions including the primary motor, somatosensory, and extra-motor areas, supporting the concept that ALS is a multi-system disorder. Specifically, our study shows an interesting finding that DC in the visual areas was altered in patients. ALS patients with higher DC in right inferior occipital gyrus have more severity of disease and the altered DC value in right inferior occipital gyrus can probably be used to assess severity of ALS.

## Author contributions

CZ: Project conception and execution; writing of the first draft. XH: Project conception, article review and critique. ML: MRI data acquisitions. XY: MRI data analysis. LC: Participants recruitment and clinical data acquisition. JH: Participants recruitment and clinical data acquisition. JZ: Project conception, article review and critique. JW: Project conception, article review and critique.

## Funding

This work is supported by the National Natural Science Foundation of China (Grant No. 81200882), Chongqing Provincial Natural Science Fund (Grant No. CSTC2011jjA0737, CSTC2012jjA10086).

### Conflict of interest statement

The authors declare that the research was conducted in the absence of any commercial or financial relationships that could be construed as a potential conflict of interest.
